# Investigation into the significant role of dermal‐epidermal interactions in skin ageing utilising a bioengineered skin construct

**DOI:** 10.1002/jcp.31463

**Published:** 2024-10-08

**Authors:** Lydia Costello, Kirsty Goncalves, Paola De Los Santos Gomez, Ben Hulette, Teresa Dicolandrea, Michael J. Flagler, Robert Isfort, John Oblong, Charlie Bascom, Stefan Przyborski

**Affiliations:** ^1^ Department of Biosciences Durham University Durham UK; ^2^ Mason Business & Innovation Center The Procter and Gamble Company Ohio USA; ^3^ Reprocell Europe Glasgow UK

**Keywords:** ageing, bioengineering, dermal‐epidermal crosstalk, extracellular matrix, human skin equivalent, inflammation

## Abstract

Increased prevalence of skin ageing is a growing concern due to an ageing global population and has both sociological and psychological implications. The use of more clinically predictive in vitro methods for dermatological research is becoming commonplace due to initiatives and the cost of clinical testing. In this study, we utilise a well‐defined and characterised bioengineered skin construct as a tool to investigate the cellular and molecular dynamics involved in skin ageing from a dermal perspective. Through incorporation of ageing fibroblasts into the dermal compartment we demonstrate the significant impact of dermal‐epidermal crosstalk on the overlying epidermal epithelium. We characterise the paracrine nature of dermal‐epidermal communication and the impact this has during skin ageing. Soluble factors, such as inflammatory cytokines released as a consequence of senescence associated secretory phenotype (SASP) from ageing fibroblasts, are known to play a pivotal role in skin ageing. Here, we demonstrate their effect on epidermal morphology and thickness, but not keratinocyte differentiation or tissue structure. Through a novel in vitro strategy utilising bioengineered tissue constructs, this study offers a unique reductionist approach to study epidermal and dermal compartments in isolation and tandem.

## Graphical abstract

An ageing dermis contributes to age‐associated changes in epidermal morphology through a paracrine mechanism. Paracrine mediators that govern this response may be released as ageing fibroblasts exhibit a Pro‐inflammatory secretome.

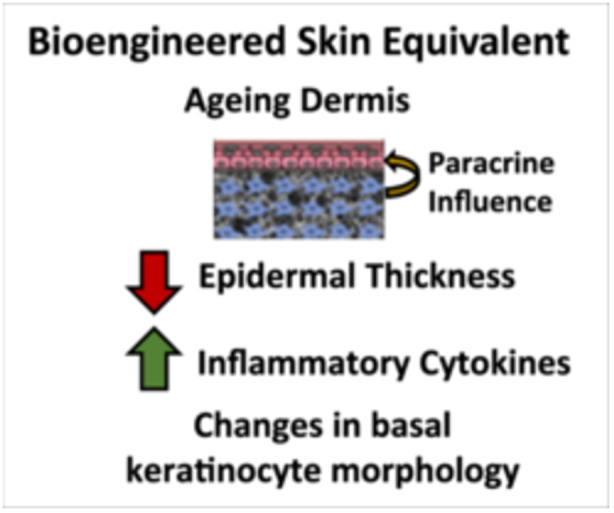

## 1 INTRODUCTION

The world has an ageing population, and the number of individuals aged 60 years or over is projected to increase by 56% to 1.4 billion by 2030 (United Nations, 2015). Ageing skin is associated with dermatological and cosmetic concerns, due to the increased prevalence of debilitating skin conditions and the psychosocial implications of changes in physical appearance (Farage et al., 2013). There is a demand to further understand the intricate molecular mechanisms that contribute to the ageing skin phenotype, including the influence of epidermal‐dermal interactions with age, to inform novel anti‐ageing interventions.

Epithelial‐mesenchymal interactions are important during embryogenesis, and it is thought that the mesoderm‐derived dermis influences epidermal histogenesis *in utero* (Kratochwil & Yamada, 1983). In addition to the early stages of life, it is thought that the dermis also plays an essential role in epidermal maintenance and morphology in adult skin and influences the ageing process. Early experimental evidence using recombinant studies in the 20th century investigated the nature of the dermal influence, and two potential mechanisms were proposed: the dermal provision of a physical substrate for the support, attachment and orientation of basal keratinocytes, and the secretion of diffusible factors from dermal cells that influence epidermal morphology (Billingham & Silvers, 1967; Wessells, 1962; Wheeler & Briggaman, 1971). These two mechanisms may not be mutually exclusive and may co‐exist.

It has also been speculated that dermal‐epidermal interactions may accelerate the ageing process, as the dermis undergoes many structural, compositional and secretory modifications which are hypothesised to affect epidermal morphology (Lee et al., 2016; Marcos‐Garcés et al., 2014; Varani et al., 2006). From a mechanistic perspective, the age‐related atrophy of the dermal extracellular matrix (ECM) may impact its role as a physical support for the epidermis (Abigail K Langton, Alessi, et al., 2019), and the alterations of the ageing fibroblast secretome including Pro‐inflammatory cytokines, pro‐oxidant reactive oxygen species and matrix‐degrading enzymes may influence keratinocyte behaviour during human skin ageing (Coppé et al., 2008, 2010; Waldera Lupa et al., 2015). Specifically, as fibroblasts age, they can undergo cell senescence and display a senescence‐associated secretory phenotype (SASP) which involves secretion of Pro‐inflammatory factors such as IL‐1, −6 and −8 (Nelson et al., 2012, 2018; Passos et al., 2010).

The impact of epidermal‐dermal crosstalk during ageing has not been extensively studied, and elucidating underlying mechanisms can be technically challenging. Tissue engineering strategies provide a valuable tool to investigate dermal‐epidermal interactions in vitro, reducing the need for animal models. Several approaches have been applied to study dermal‐epidermal crosstalk during ageing including the inclusion of ageing and senescent fibroblasts or advanced glycation end products (AGEs) within human skin equivalents (HSEs) (Costello et al., 2022). These studies demonstrated that an ageing dermis affected epidermal morphology; however, the underlying mechanisms were not explored (Hausmann et al., 2019; Lacroix et al., 2007). HSEs provide a unique platform capable of providing mechanistic insights and hypotheses, through studying the direct Coculture of epidermal and dermal compartments in a full thickness HSE (FT‐HSE), or indirect contact through conditioned media studies on isolated compartments.

In this study, we describe the application of a well‐established, characterised and published HSE that has previously been applied to many research topics in the field of skin health. Herein, we have applied this versatile model to investigate crosstalk during ageing through Coculture of neonatal keratinocytes with ageing fibroblasts within the FT‐HSE environment, which resulted in significant morphological disparities. We demonstrate that the differences in epidermal morphology are induced in a paracrine manner mediated by diffusible factors secreted by ageing fibroblasts through bioengineering of the epidermal compartment in isolation. This technology provides a platform to probe the underlying molecular mechanisms that underpin dermal‐epidermal crosstalk whilst also lending itself to the identification of interventions, screening of actives or testing of dermatological procedures for their ability to attenuate aspects of the ageing epidermal phenotype.

## 2 MATERIALS & METHODS

### 2.1 Full thickness human skin equivalent generation

FT‐HSEs were constructed as previously described (Costello, Goncalves, Maltman, et al., 2023; Freer et al., 2023; Goncalves et al., 2023; Roger et al., 2019). Human neonatal dermal fibroblasts (HDFn, Lot #1366356, Thermo Fisher Scientific, Loughborough, UK) and human adult dermal fibroblasts from a 60‐year‐old individual (HDFa, Lot #1090465, ThermoFisher Scientific) were incorporated into the dermal compartment. Briefly, 0.5 × 10^6^ HDFn or HDFa were seeded into 12 well Alvetex® Scaffold inserts (Reprocell Europe Ltd, Glasgow, UK), a porous, inert, polystyrene scaffold. They were then cultured in Medium 106® (ThermoFisher Scientific) supplemented with 5 ng.mL^−1^ transforming growth factor β1 (TGFβ1) and 100 μg.mL^−1^ ascorbic acid. Following 30 days in culture, neonatal human epidermal keratinocytes (HEKn Lot #1366356, ThermoFisher Scientific) were seeded onto dermal compartments to form the epidermis. HSEs were cultured in submerged conditions for 48 h in EpiLife® Medium supplemented with 10 ng.mL^−1^ keratinocyte growth factor (KGF), 160 μM CaCl_2_ and 100 μg.mL^−1^ ascorbic acid. HSEs were then raised to the air‐liquid interface (ALI) and cultured in EpiLife® Medium supplemented with 10 ng.mL^−1^ KGF, 1.64 mM CaCl_2_ and 100 μg.mL^−1^ ascorbic acid to promote keratinocyte differentiation. FT‐HSEs were cultured to 14 days ALI before analysis.

### 2.2 Epidermal only skin equivalent generation

Epidermal‐only skin equivalents (EO‐HSEs) were generated as previously described (Bjerke et al., 2021; Roger et al., 2019). Briefly, 0.5 × 10^6^ HEKn were seeded onto collagen‐coated Millicell® cell culture inserts (Merck Millipore, Beeston, UK) and cultured in Epilife® Medium supplemented with 5 ng.mL^−1^ KGF, 160 μM CaCl_2_ and 50 μg.mL^−1^ ascorbic acid for 48 h in submerged culture. EO‐HSEs were then raised to the ALI and cultured in Epilife® Medium supplemented with 5 ng.mL^−1^ KGF, 1.64 mM CaCl_2_ and 50 μg.mL^−1^ ascorbic acid to promote keratinocyte differentiation. EO‐HSEs were maintained for 7 days to form mature constructs.

Fibroblast conditioned medium was obtained through culture of confluent HDFn and HDFa populations adjusted to give a consistent cell density, in Epilife® Medium for 24 h. EO‐HSEs were then cultured in conditioned media from neonatal or ageing fibroblasts for a further 7 days before analysis. The conditioned medium was mixed 1:1 with fresh Epilife® and supplemented with 5 ng.mL^−1^ KGF, 1.64 mM CaCl_2_ and 50 μg.mL^−1^ ascorbic acid.

### 2.3 Wax embedding and sectioning

Skin equivalents were fixed in 4% paraformaldehyde (Sigma‐Aldrich, Missouri, US) then dehydrated from 30% to 100% ethanol (v/v). Samples were incubated in Histo‐Clear (Scientific Laboratory Supplies, Nottingham, UK) for 30 min, a 1:1 ratio of Histo‐Clear and paraffin wax (Thermo Fisher Scientific) for 30 min at 65°C followed by paraffin wax alone for 1 h at 65°C before embedding in plastic moulds. The samples were sectioned at 5 μm using a microtome (Leica, Wetzlar, Germany) and mounted onto charged microscope slides (Thermo Fisher Scientific).

### 2.4 Histological staining

Samples were deparaffinised in Histo‐Clear, rehydrated from 100% ethanol to distilled water (dH_2_O) then stained with Mayer's haematoxylin (Sigma‐Aldrich) for 5 min. Sections were washed in dH_2_O, transferred to alkaline alcohol for 30 s then dehydrated from 70% to 95% ethanol (v/v). Samples were then stained in eosin (Sigma‐Aldrich) for 30 s, further dehydrated in 95% and 100% ethanol (v/v), then incubated in Histo‐Clear before mounting with Omni‐mount (Scientific Laboratory Supplies). Samples were imaged using a light microscope and the Leica EZ software (Leica).

### 2.5 Immunofluorescence staining

Samples were de‐paraffinised in Histo‐Clear, then rehydrated from 100% ethanol to phosphate buffered saline (PBS). Antigen retrieval was performed in citrate buffer (pH 6, Sigma‐Aldrich) at 95°C for 20 min. After cooling, samples were incubated with blocking solution (20% neonatal calf serum in 0.4% Triton X‐100 in PBS) for 1 h at room temperature. Primary antibodies (Table S1) diluted 1:100 in the blocking solution were added to the samples and incubated overnight at 4°C. Samples were washed in PBS and incubated with the secondary antibodies (donkey anti‐mouse Alexa Fluor® 488 A21202; donkey anti‐rabbit Alexa Fluor® 594 A21207, donkey anti‐rabbit Alexa Fluor® 488 A‐21206 Thermo Fisher Scientific) at a 1:1000 dilution in blocking buffer for 1 h at room temperature. Samples were washed in PBS before mounting in Vectashield Hardset with DAPI (Vector Laboratories, Peterborough, UK). Images were captured using a Zeiss 880 confocal microscope with Zen software.

### 2.6 Biometric quantification

All biometric quantification was conducted using freely available Image J software (imagej. net) (Schneider et al., 2012).

#### 2.6.1 Epidermal thickness

Viable epidermal thickness was measured as previously described (Bjerke et al., 2021; Costello, Goncalves, De Los Santos Gomez, et al., 2023; Freer et al., 2023) from the basolateral edge of the basal keratinocytes to the apical surface of the *stratum granulosum* from H&E‐stained images using the straight‐line tool in the Image J software with the appropriate scale.

#### 2.6.2 Number of viable epidermal layers

Immunofluorescent images depicting K10 and K14 reactivity were quantified using the multipoint tool in Image J to count the number of viable epidermal cell layers from the *stratum basale* to the *stratum granulosum*.

#### 2.6.3 Percentage epidermal cells

Immunofluorescence‐stained images for p63, Ki67 and HMGB1 represent extent of epidermal stemness, proliferation and senescence respectively. In all cases the multipoint tool in Image J was used to quantify positive staining for each antigen and total viable cell count (DAPI staining), expression was then calculated as a percentage of total cells.

#### 2.6.4 Basal keratinocyte morphology

The area, height and width of basal keratinocytes was quantified using the polygon and line tools in Image J software from K10 and K14 stained immunofluorescence images, as previously described (Costello, Goncalves, De Los Santos Gomez, et al., 2023). The height and width measurements were used to calculate the height:width ratio of basal keratinocytes.

### 2.7 Cytokine array

Samples of culture medium were harvested from either neonatal or ageing fibroblast populations. Analysis of the cytokine content of the medium was performed by Eve Technologies (Calgary, CA) and Human Cytokine Pro‐inflammatory Focused 15‐Plex Discovery Assay® Array (HDF15) was conducted.

### 2.8 Graphs and statistics

Graphs are expressed as mean ± standard error of the mean (SEM). All statistical analyses were performed via GraphPad Prism 5 software using an unpaired, two‐tailed t‐test with Welch's correction. Differences between the groups were considered significant when *p* < 0.05, and the significance is depicted graphically for each data set where *p < 0.05, **p < 0.01, ***p < 0.001, ****p < 0.0001, not significant (ns) *p* > 0.05.

## 3 RESULTS

### 3.1 Dermal compartments containing ageing fibroblasts exhibit hallmarks of ageing

**Figure 1:**
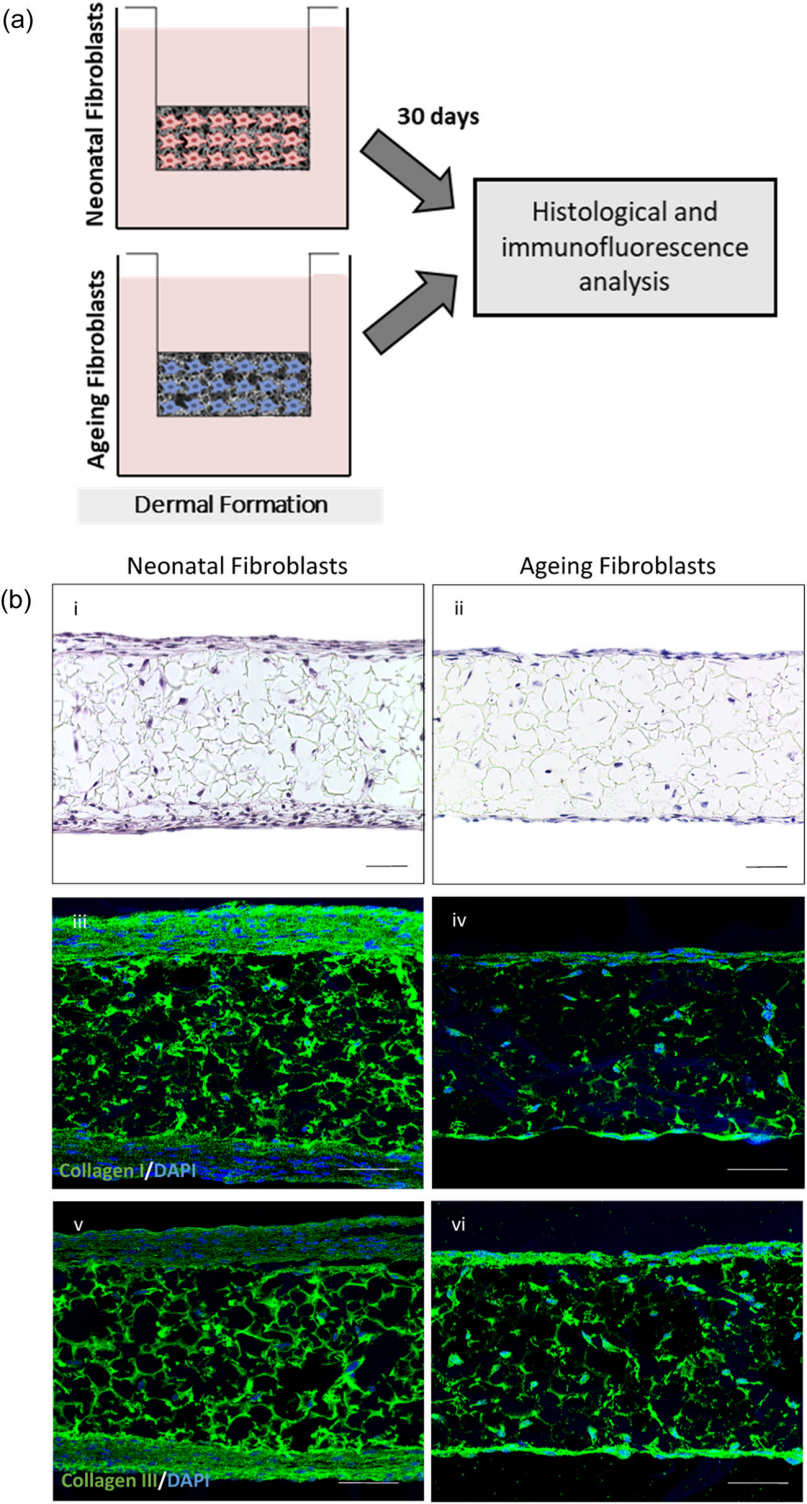
Ageing dermal compartments exhibit decreased extracellular matrix deposition. Neonatal or ageing fibroblasts were seeded in Alvetex® Scaffold and cultured for 30 days before being harvested for analysis (a). H&E staining of dermal compartments generated with neonatal (bi) and ageing fibroblasts (bii) demonstrate fibroblast population of the scaffold. Representative immunofluorescence staining of collagen I (biii) and collagen III (biv) is reduced in ageing dermal compartments. Collagens are stained green and nuclei are stained with DAPI. Scale bars: B = 50 μm.

Commercially available fibroblasts from either a neonatal or ageing donor were seeded into Alvetex® Scaffold and cultured for 30 days before analysis (Figure 1a). Histological analysis (Figure 1bi,ii) revealed dermal equivalents comprising of ageing fibroblasts contained fewer fibroblasts within and around the peripheries of the scaffold, thought to be a consequence of reduced proliferation. Immunofluorescence analysis of dermal compartments containing either neonatal or ageing fibroblasts, demonstrated the ability of both fibroblast populations to produce endogenous ECM such as collagen I (Figure 1biii,iv) and collagen III (Figure 1bv,vi) within the scaffold. However, ageing dermal compartments contained significantly less of both collagens compared with their neonatal counterpart, consistent with dermal ageing in vivo (Abigail Kate Langton, Graham, et al., 2019).

### 3.2 Coculture of an ageing dermal compartment with a neonatal epidermis, significantly impacts epidermal thickness and basal keratinocyte morphology

**Figure 2:**
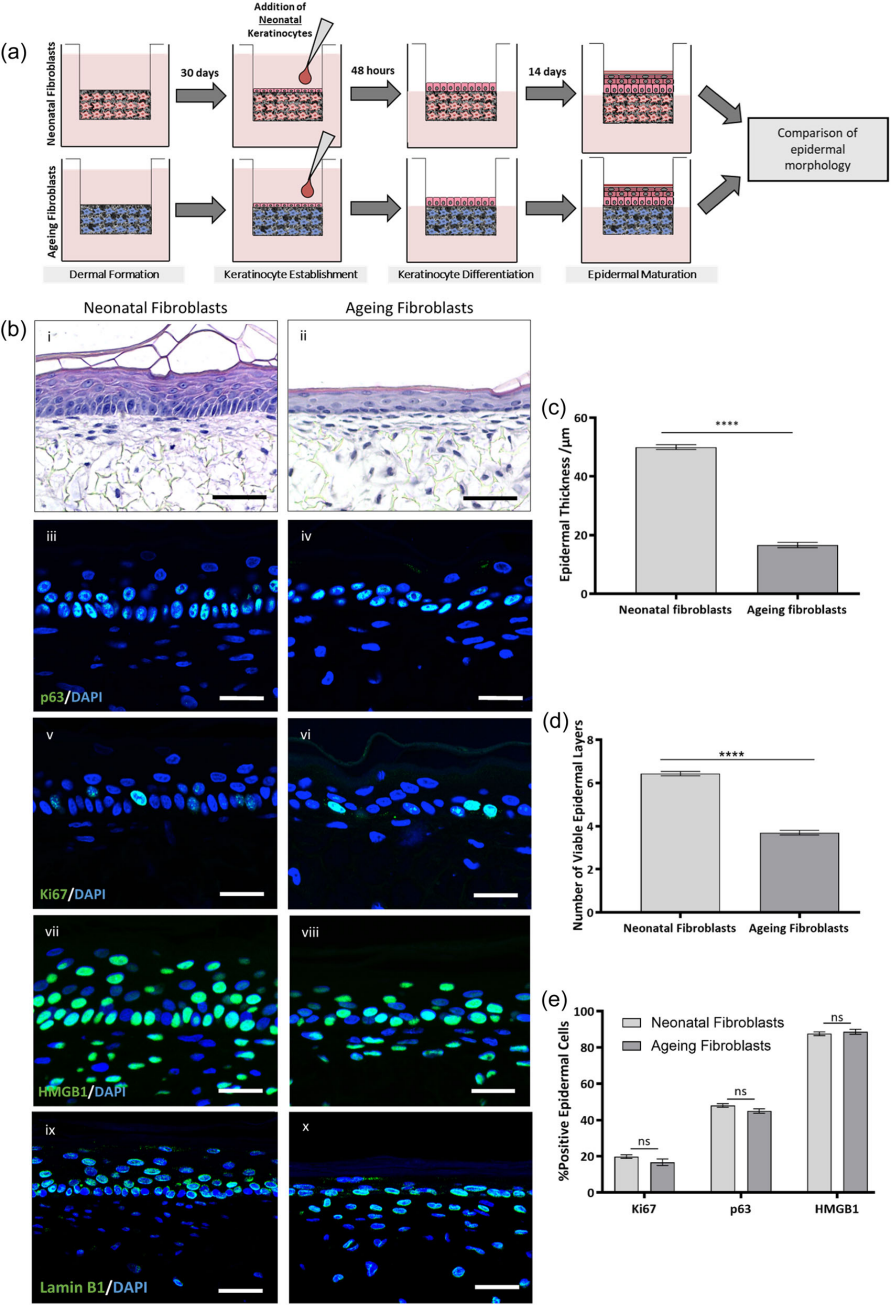
Coculture of an ageing dermal compartment with neonatal keratinocytes results in epidermal thinning independent of keratinocyte proliferation. Dermal compartments were generated using both neonatal and ageing fibroblast populations, upon which, neonatal keratinocytes were seeded and allowed to stratify at the air‐liquid interface for 14 days before analysis (a). Representative H&E stained images display significant epidermal thinning upon an ageing dermis (bi, ii). Immunofluorescence staining of proliferation, stemness and senescence‐associated biomarkers (green): p63 (biii, iv), Ki67 (Bv‐vi), HMGB1 (vii‐viii) and Lamin b1 (bix,x). Nuclei are stained in blue. Quantification of epidermal thickness (c) and number of viable epidermal layers (d) are significantly reduced in the presence of an ageing dermal compartment. Whereas, the percentage of ki67, p63 and HMGB1 remains unchanged regardless of dermal foundation (e). Data represent mean ± SEM, *n* = 9 and a two‐tailed, unpaired *t*‐test with Welch's correction was performed where: ****p < 0.0001 and *p* > 0.05 is nonsignificant. Scale bars: Bi, ii = 50 μm, Biii‐x = 25 μm.

To determine the impact of an ageing dermis on the overlying epidermal epithelium in vitro, neonatal keratinocytes were cocultured onto a mature dermis containing either neonatal or ageing fibroblasts, allowed to proliferate in submerged cultured, followed by stratification at ALI, then harvested for analysis (Figure 2a). The resultant tissue construct was a FT‐HSE containing either neonatal or ageing fibroblasts within the dermal compartment and an overlying epidermis comprising of neonatal keratinocytes.

Upon histological examination (Figure 2bi,ii), it was evident that both a neonatal and ageing dermis was able to support an overlying epidermis, with no evidence of keratinocyte invasion into the dermal compartment. Epidermal compartments in both conditions appeared differentiated, stratified and keratinised. Despite the formation of an organised epidermis, a significant 67% decrease in epidermal thickness was observed when cocultured with an ageing dermis (*p* < 0.0001) (Figure 2c). This was accompanied by a significant reduction in the number of viable epidermal layers (*p* < 0.0001) (Figure 2d) in the presence of an ageing dermis. We hypothesised that this could be due to reduced stemness, proliferative capacity or increased senescence of keratinocytes, as a consequence of the bystander effect induced by ageing fibroblasts. However, immunofluorescence staining for p63 (Figure 2biii,iv), an essential transcription factor for the development of stratified epithelia, and Ki67 (Figure 2bb,vi), a well‐defined marker of proliferation resulted in no significant differences in epidermal expression despite the presence of an ageing dermis (*p* > 0.05) (Figure 2e). Similarly, immunofluorescence staining for the senescence‐associated biomarker: HMGB1 (Figure 2bvii,viii), which is reported to translocate from the nucleus to the cytoplasm in senescent cells, was also found to not differ significantly in either expression or localisation when keratinocytes were cocultured with an ageing dermis (*p* > 0.05) (Figure 2e). This observation was also supported by no significant difference in Lamin B1 staining (Figure 2bix,x), another marker of cellular senescence.

**Figure 3:**
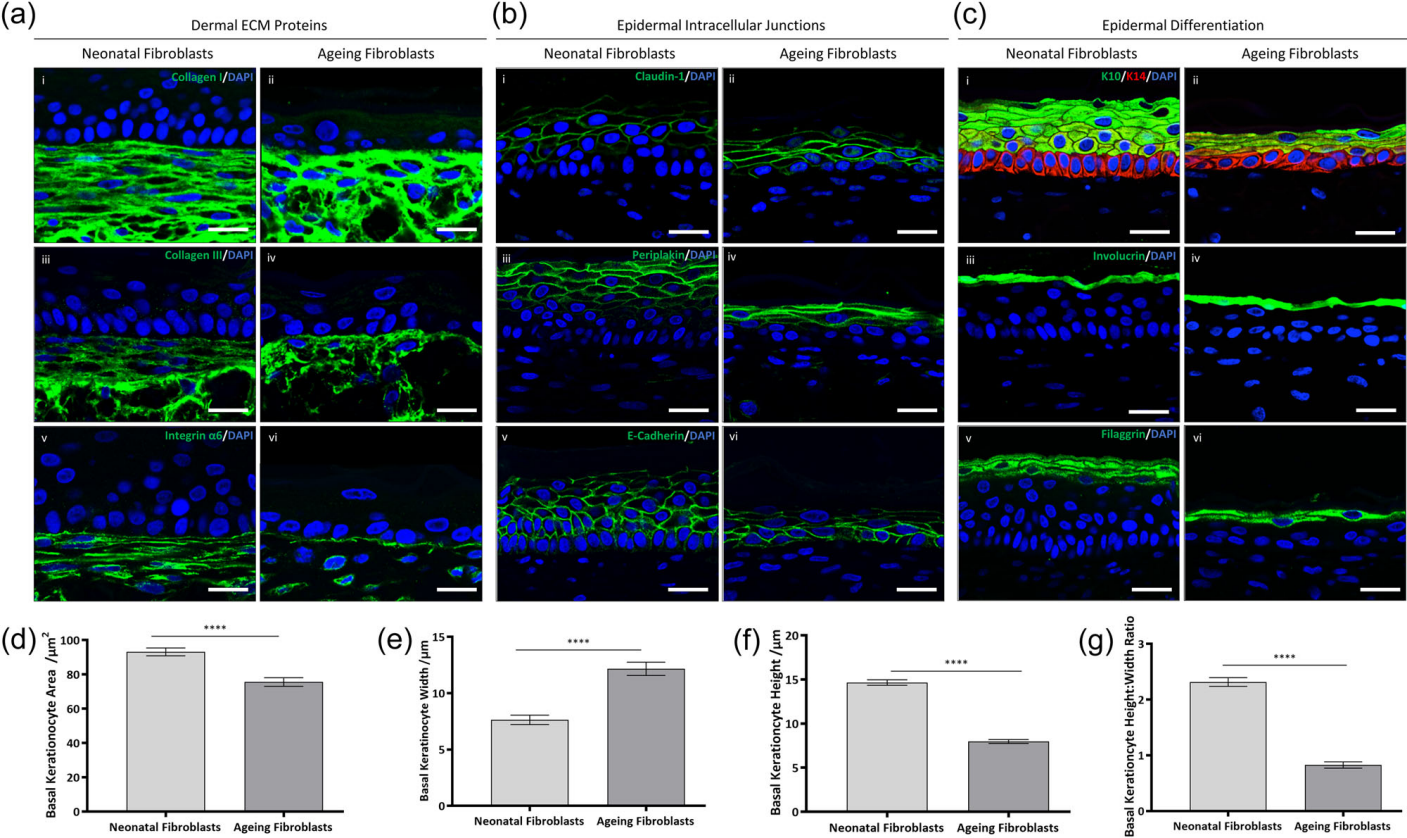
Coculture of ageing fibroblasts with neonatal keratinocytes significantly impacted keratinocyte morphology but not differentiation or intracellular junctions. Representative immunofluorescence images of dermal‐associated extracellular matrix (ECM) proteins (a): collagen I (ai, ii), collagen III (aiii, iv) and integrin α6 (av, vi), stained in green. Immunofluorescence images of epidermal intracellular junctional biomarkers (b): claudin‐1 (bi, ii), periplakin (biii, iv) and E‐cadherin (bv, vi), stained in green. Representative immunofluorescence analysis of biomarkers associated with epidermal differentiation (c) including: keratin‐10 (green) and keratin‐14 (red, ci, ii), involucrin (ciii, iv) and filaggrin (cv, vi) stained in green. In all immunofluorescence images nuclei are stained with DAPI in blue. Biometrics quantification of aspects of basal keratinocyte morphology including area (d), width (e), height (f) and height:width ratio (g) are significantly impacted in the presence of an ageing dermis. Data represent mean ± SEM, *n* = 9 and a two‐tailed, unpaired *t*‐test with Welch's correction was performed where ****p < 0.0001. Scale bars: 25 μm.

Despite the striking effects of an ageing dermis on epidermal thickness, little impact on epidermal structure or keratinocyte differentiation was observed. To better understand the structural impact of an ageing dermis on the overlying epidermis, a series of immunofluorescent analyses were performed for biomarkers specific to dermal ECM proteins (Figure 3a), epidermal intracellular junctions (Figure 3b) and epidermal differentiation (Figure 3c). Both collagen I and III were found to be abundant in both neonatal and ageing dermal compartments; however, they appeared less organised and at reduced levels in ageing dermal compartments. Integrin α6, a biomarker of the basement membrane was detectable in both neonatal and ageing conditions, suggesting that an ageing dermis is not detrimental to basement membrane formation in this FT‐HSE system.

Despite a significant reduction in epidermal thickness when cultured on an ageing dermal foundation, several biomarkers related to intracellular junctions were identifiable in both neonatal and ageing dermis‐based FT‐HSEs. Positive staining for claudin‐1, periplakin and E‐cadherin was evident in epidermises cultured on both neonatal and ageing‐fibroblast based dermal compartments. This suggests, despite differences in epidermal morphology, communication and close interactions between keratinocytes remained unaffected. Furthermore, expression of biomarkers specific to epidermal differentiation were investigated. Keratin‐14 positive basal keratinocytes were identifiable in both epidermises cultured on neonatal and ageing dermal compartments, with suprabasal keratin‐10 positive keratinocytes also evident. Similarly, expression of biomarkers consistent with terminal keratinocyte differentiation: involucrin and filaggrin. Together these data suggest there is no impact on keratinocyte differentiation by incorporation of an ageing dermal compartment in this model system, and although the epidermis is thinner, it remains structurally intact.

Our data suggested that there were no identifiable differences in stemness, proliferation and senescence via immunofluorescence analysis. This suggests that the impact of an ageing dermal population on a neonatal epidermal population results in a solely morphological change. Therefore, we hypothesised that the changes in epidermal structure could be driven by changes in morphology of the keratinocytes themselves. This was evident in the immunofluorescence staining of keratin‐14 (Figure 3ci,ii), where keratinocytes in the *stratum basale* appeared to adopt a different morphology when cocultured with ageing fibroblasts. Biometric quantification of basal keratinocyte morphology supported this observation with significantly reduced area (*p* < 0.0001) (d) and height (*p* < 0.0001) (f) along with increased with (*p* < 0.0001) (e), which correlated to a significantly decreased height:width ratio (*p* < 0.0001) (g). Height:width ratio when cultured with an ageing dermal compartment, decreased from 2.3 to 1, which corresponds with basal keratinocytes adopting a more cuboidal morphology compared with their characteristic columnar morphology observed in the presence of a neonatal dermal compartment. Cytokeratin 10 staining also highlights potential differences in the suprabasal layer formation, as many keratinocytes become anuclear after delamination from the *stratum basale* when cocultured with an ageing dermis.

These data suggest that the presence of an ageing dermis impacts epidermal morphology through communication with the overlying neonatal epithelium and is capable of induction of significant alterations at a cellular level. To investigate the mechanism of action further, we determined the role of paracrine mediators as signals between the two compartments by undertaking a series of conditioned medium experiments.

### 3.3 An ageing dermis influences epidermal morphology in a paracrine fashion through release of pro‐inflammatory mediators

Morphological differences in epidermal structure were observed when cocultured with ageing fibroblasts in the full thickness skin equivalents (FT‐HSEs). Although more physiologically representative of the native tissue, the complexity of the FT‐HSEs do not allow for the intricacies of single pathways/mechanisms to be fully identified and explored. This is due to the fact that within these FT‐HSE constructs, the fibroblasts are in direct contact with the keratinocytes, and once maturation of the tissue is established are separated only by the basement membrane assembled de novo by the cells themselves, therefore a clear distinction between the effects of cell‐cell contact, physical support of the dermis and the influence of diffusible factors is not possible. We hypothesised that the observed differences were due to the paracrine effect of diffusible factors, and this was investigated through application of EO‐HSEs.

**Figure 4:**
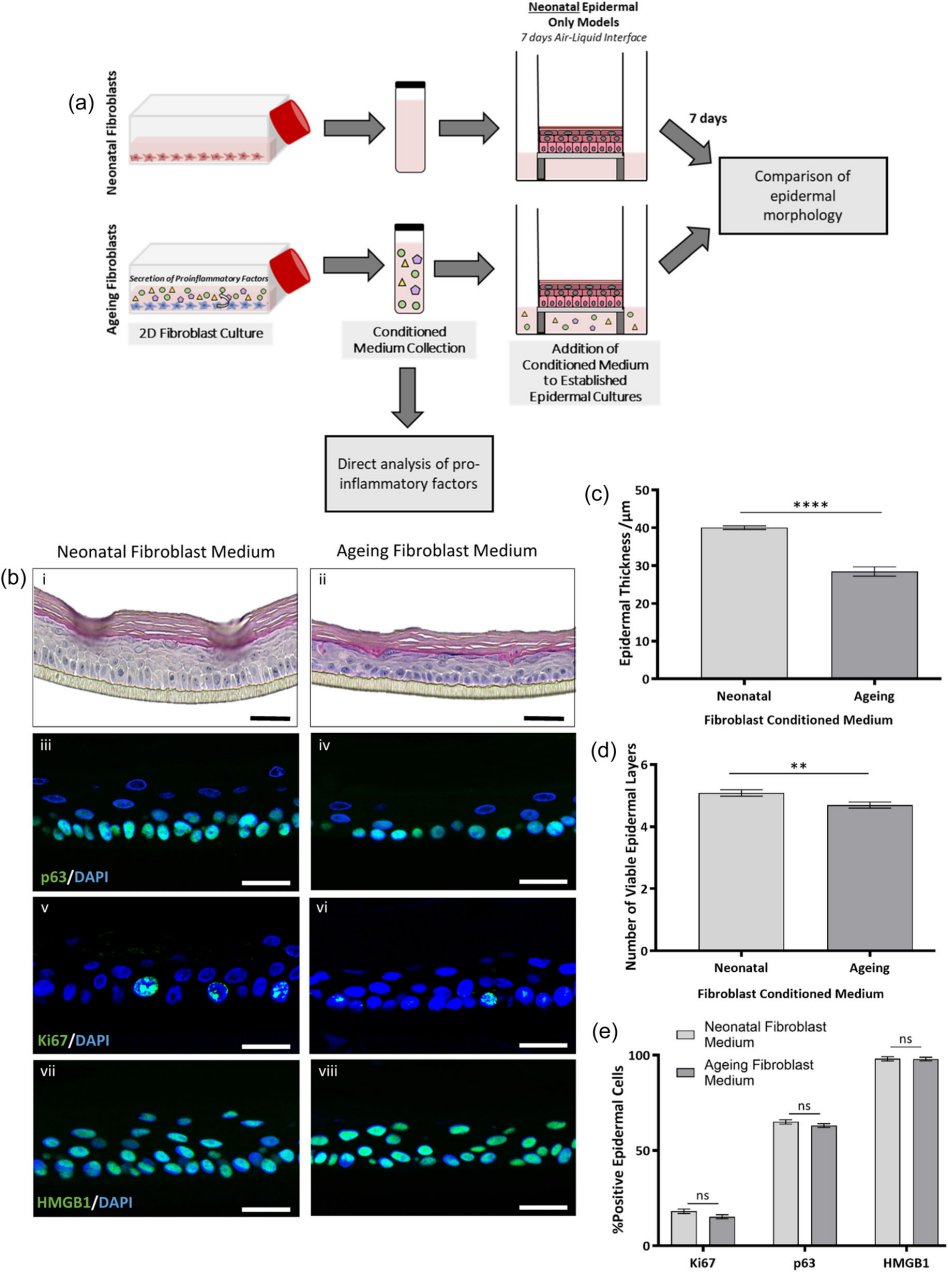
Ageing fibroblast conditioned medium significantly impacts epidermal morphology in a monoculture epidermal construct, independent of keratinocyte proliferation. Populations of neonatal or ageing fibroblasts were expanded in 2D culture and samples of their conditioned medium obtained for analysis or application to tissue constructs. Epidermal‐only human skin equivalents (EO‐HSEs) containing neonatal keratinocytes were cultured for 7 days at the air‐liquid interface to form a mature epidermis before fibroblast conditioned medium was added to the constructs for a further 7 days before analysis (a). (a) Representative H&E staining of EO‐HSEs cultured in neonatal or fibroblast conditioned medium (bi, ii). Representative immunofluorescence staining of biomarkers (green): p63 (biii, iv), ki67 (bv, vi) and HMGB1 (bvii, viii). Nuclei are stained in blue by DAPI. Biometric analysis of epidermal thickness (c) and number of viable epidermal layers (d) reveal significant differences between the two conditioned media types. Whereas, quantification of percentage positive epidermal cells of ki67, p63 and HMGB1 (e) reveals no significant difference between conditions. Data represent mean values ± SEM, *n* = 9 and a two‐tailed, unpaired *t*‐test with Welch's correction was performed where **p < 0.01, ****p < 0.0001 and *p* > 0.05 is nonsignificant. Scale bars: bi, ii = 50 μm, biii‐viii = 25 μm.

To investigate the role of soluble mediators and paracrine action in crosstalk between epidermal and dermal compartments, conditioned medium from both neonatal and ageing fibroblast populations was collected. This medium was then either harvested for analysis or mixed in at 1:1 with fresh cultured medium and added to established EO‐HSEs derived from neonatal keratinocytes (Figure 4a). EO‐HSEs were cultured to 7 days ALI to allow for epidermal maturation and stratification to occur, fibroblast conditioned medium was then added to the EO‐HSEs, which were further cultured for 7 days before analysis.

Histological analysis (Figure 4bi,ii) revealed well‐structured and viable epidermal constructs in the presence of both neonatal and ageing fibroblast conditioned medium, although as with previous FT‐HSE studies, the difference in epidermal thickness was striking. Quantification of epidermal thickness (Figure 4c) supported this observation as conditioned medium from ageing fibroblasts induced a significant 29% decrease in epidermal thickness (*p* < 0.0001). This decreased epidermal thickness was accompanied by a small, but significant decrease in number of viable epidermal layers (*p* < 0.01) (Figure 4d). This suggests that the observed thinning of the epidermis could be attributed, in part, to diffusible factors secreted from ageing fibroblasts.

Similarly to FT‐HSE study, this decrease in epidermal thickness appeared to be independent of stemness, proliferation, or senescence as no significant differences were observed in the percentage of p63‐ (Figure 4biii,iv), Ki67‐ (Figure 4v,vi) or HMGB1‐positive (Figure 4vii,viii) nuclei when cultured in neonatal or ageing fibroblast conditioned media (*p* > 0.05) (Figure 4e).

**Figure 5:**
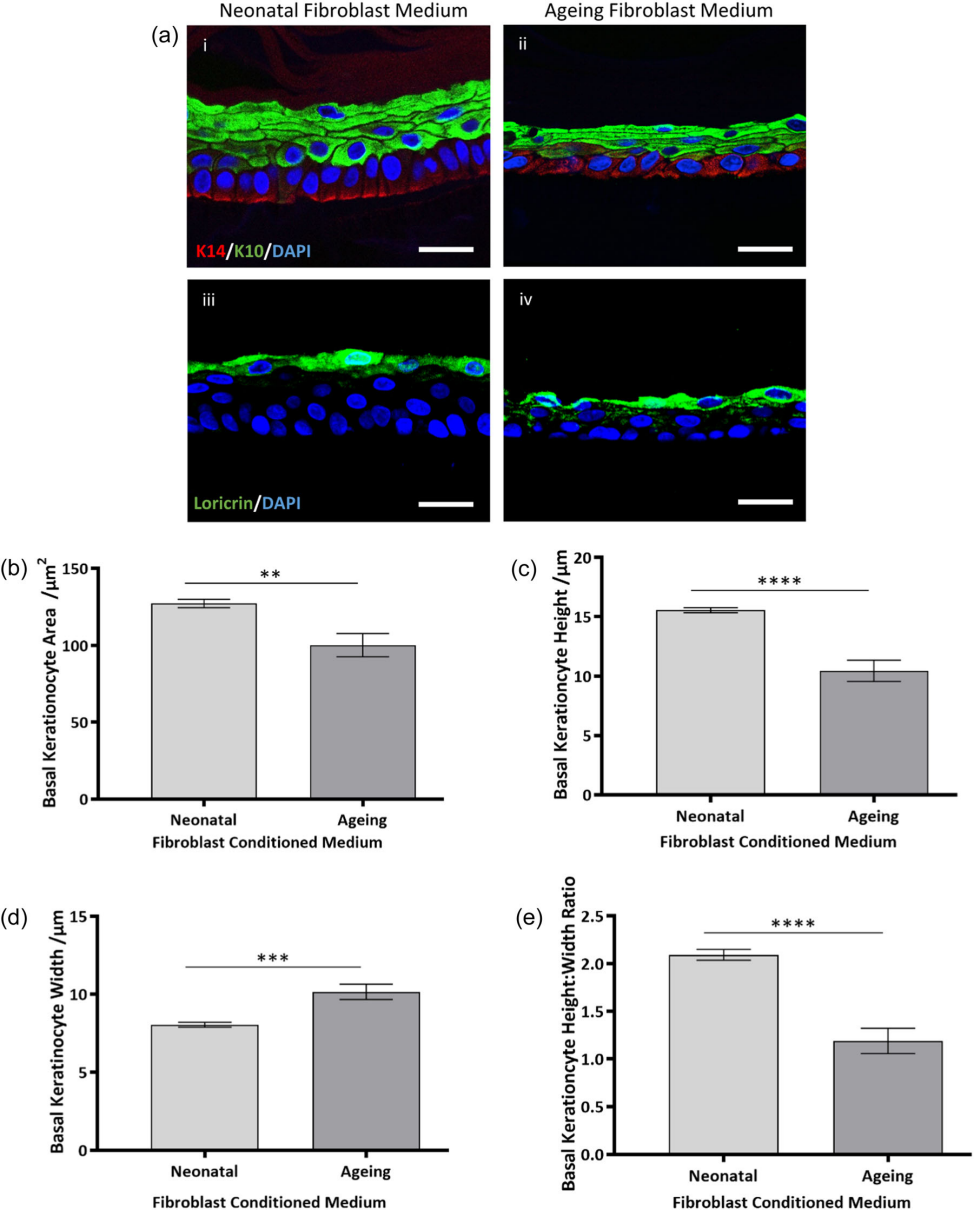
Ageing fibroblast conditioned medium significantly impacts basal keratinocyte morphology but not differentiation and stratification of neonatal keratinocytes in a monoculture epidermal construct. Representative immunofluorescence images of epidermal‐only HSEs (EO‐HSEs) cultured in medium conditioned by either neonatal or ageing fibroblasts and stained for keratinocyte differentiation related biomarkers: keratin‐10 (green) and keratin‐14 (red, ai, ii), and loricrin (aiii, iv). Nuclei are stained by DAPI in blue. Quantification of aspects of basal keratinocyte morphology reveal significant differences, including: area (b), height (c), width (d) and height:width ratio (e). Data represent mean values ± SEM, *n* = 9 and a two‐tailed, unpaired *t*‐test with Welch's correction was performed where **p < 0.01, ***p < 0.001, ****p < 0.0001. Scale bars: 25 μm.

EO‐HSEs cultured in conditioned medium from neonatal and ageing fibroblasts exhibit an organised epidermis with evidence of early‐ (keratin‐14, Figure 5ai,ii), mid‐ (keratin‐10, Figure 5ai,ii) and terminal differentiation (loricrin, Figure 5aiii,iv). Similarly to the direct coculture within FT‐HSEs, differences in basal keratinocyte morphology were identified by keratin‐14 staining. Biometric quantification revealed differences in basal keratinocyte morphology through the addition of ageing fibroblast conditioned medium to EO‐HSEs. Basal keratinocytes were demonstrated to be reduced in area (*p* < 0.01) (Figure 5b), reduced in height (*p* < 0.0001) (Figure 5c), increased in width (*p* < 0.001) (Figure 5d) and decreased in height:width ratio from 2.1 to 1.2 (*p* < 0.0001) (Figure 5e), which is indicative of a more cuboidal morphology. These observations reflect the full thickness skin coculture experiments, which suggests that diffusible factors from ageing fibroblasts may, in part, induce structural changes to the basal keratinocytes.

**Figure 6:**
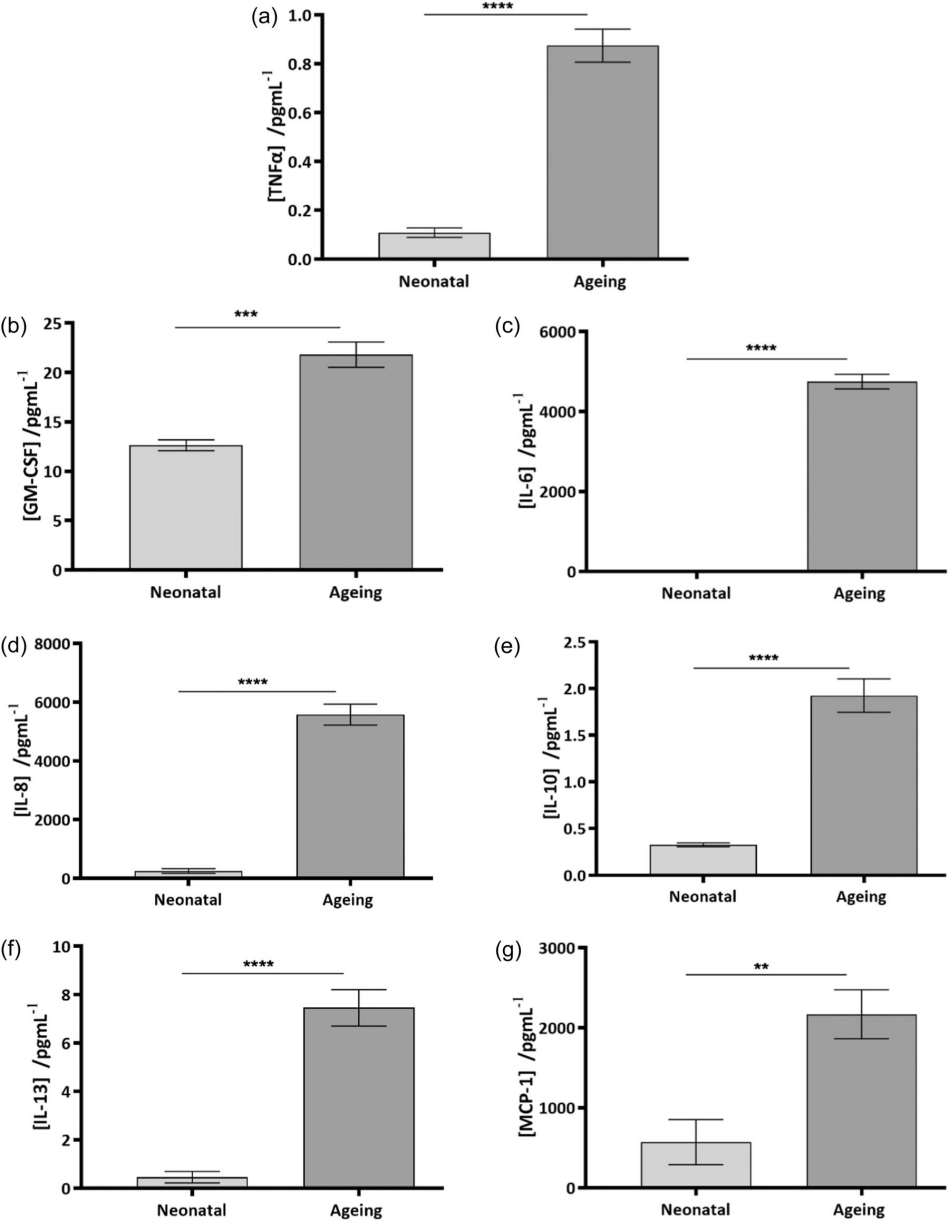
Ageing fibroblasts exhibit a more pro‐inflammatory secretome as opposed to neonatal fibroblasts. A significant increase in the concentration of pro‐inflammatory cytokine release was observed from medium conditioned by ageing fibroblasts. Concentrations of cytokines such as: TNF‐α (a), GM‐CSF (b), IL‐6 (c), IL‐8 (d), IL‐10 (e), IL‐13 (f) and MCP‐1 (g) were all significantly increased in conditioned medium collected from ageing fibroblast populations. Data represent mean values ± SEM, *n* = 6 and a two‐tailed, unpaired *t*‐test with Welch's correction was performed where **p < 0.01, ***p < 0.001, ****p < 0.0001.

To better understand the composition of the conditioned medium and speculate which paracrine mediators may have instigated the epidermal morphological changes observed, expression analysis of a panel of a pro‐inflammatory cytokines was conducted on samples of conditioned medium harvested from both neonatal and ageing fibroblast populations. This analysis revealed a number of cytokines to be present in significantly higher concentrations in ageing fibroblast conditioned medium such as: TNF‐α (*p* < 0.0001) (Figure 6a), GM‐CSF (*p* < 0.001) (Figure 6b), IL‐6 (*p* < 0.0001) (Figure 6c), IL‐8 (*p* < 0.0001) (Figure 6d), IL‐10 (*p* < 0.0001) (Figure 6e), IL‐13 (*p* < 0.0001) (Figure 6f) and MCP‐1 (*p* < 0.01) (Figure 6g). These findings support the notion that as cells age, their secretome becomes more inflammatory.

## 4 DISCUSSION

Epithelial‐mesenchymal interactions are important for organogenesis of many organs and influential in the maintenance of the human dermis and epidermis in adult skin (Wheeler & Briggaman, 1971). Studies have demonstrated that dermal fibroblasts affect the formation of the basement membrane, and the viability, differentiation and thickness of the epidermis (Coulomb et al., 1989; Mackenzie & Fusenig, 1983). As alterations in the dermal biomechanical properties and secretion of diffusible factors are known to be hallmarks of the ageing process, it is hypothesised that dermal changes may impact epidermal morphology. There is increasing evidence that the age‐related epidermal morphology is, in part, due to the ageing dermis (Quan, 2023).

Previous studies suggest multiple mechanisms of how an ageing dermis can drive changes in epidermal morphology, such as: physical support, basement membrane interactions and secretion of diffusible factors. Several diffusible factors secreted by fibroblasts have been demonstrated to impact epidermal morphogenesis such as KGF (Maas‐Szabowski et al., 1999) and granulocyte‐macrophage colony stimulating factor (GM‐CSF) (Szabowski et al., 2000) however, dermal‐epidermal interactions have not been extensively described within the context of ageing.

Tissue engineered skin equivalents provide a robust in vitro platform to study these underpinning molecular mechanisms of ageing. Particularly as they offer a flexible platform that can be tailored to investigate specific aspects of dermal‐epidermal interactions. Additionally, isolation of epidermal and dermal compartments, offer a simplified, reductionist approach to probe for specific mechanistic insights. Here, we describe the application of a well‐established, characterised, FT‐HSE that recapitulates many aspects of human skin biology such as endogenous ECM secretion, basement membrane formation and intracellular junctions (Costello et al., 2019; Roger et al., 2019). We apply this model system, which has previously been adapted to study many aspects of skin health (pigmentation (Goncalves et al., 2023), neurosensory irritation (Freer et al., 2023), active screening (Bjerke et al., 2021) and exfoliation (Costello, Goncalves, Maltman, et al., 2023)), to better elucidate the molecular mechanisms that underpin paracrine communication between the dermal and epidermal compartments during the ageing process.

The use of bioengineered skin constructs in this study has allowed us to investigate the impact of dermal‐epidermal interactions during skin ageing through both direct and indirect settings. Direct coculture experiments utilising FT‐HSEs demonstrate that an ageing dermis decreases epidermal thickness, which correlates with reported epidermal atrophy with age in vivo (Pennacchi et al., 2015). In this culture model, fibroblasts are in direct contact with the keratinocytes, and therefore a clear distinction between the effects of cell‐cell contact, the physical support of the dermis and diffusible factors was not feasible. To investigate the underlying molecular mechanisms, the two compartments were spatially separated, through combination of an EO‐HSE and conditioned media from neonatal or ageing fibroblasts. This allowed the paracrine effect of fibroblasts on the epidermis to be investigated in isolation, and interestingly produced the same striking reduction of epidermal thickness in the presence of ageing fibroblast conditioned medium.

Previously, we have demonstrated the morphological changes that occur within the epidermis in vivo following both intrinsic and extrinsic ageing. This included a significant reduction in epidermal thickness (E_max_) in ageing samples from a photoprotected area, accompanied by no significant change in Ki67 expression regardless of photoexposure or age (Costello, Goncalves, De Los Santos Gomez, et al., 2023). Our findings described in this study utilising in vitro methods are consistent with previous in vivo findings, therefore validating and supporting our conclusions. Changes in basal keratinocyte morphology have also been linked to photoexposure in ageing populations, with photoexposure being the greatest environmental driver of the visible signs of ageing (Costello, Goncalves, De Los Santos Gomez, et al., 2023). Interestingly, in this study, we found that both in direct coculture and in the presence of conditioned medium from ageing fibroblasts, basal keratinocytes adopted a more cuboidal rather than distinctive columnar morphology consistent with in vivo findings (Costello, Goncalves, De Los Santos Gomez, et al., 2023). As cell shape is inherently linked to gene expression and ultimately cell function, through the process of mechanotransduction (Allcock et al., 2023), combined with the important role of basal keratinocytes in epidermal homeostasis, it is highly likely this observation may result in functional deficits.

Although outside the scope of this study, previously we have demonstrated stark differences in rete ridge and dermal papilla morphology in aged skin biopsies, and postulated this to be one of the driving factors that contributes to an ageing phenotype in vivo (Costello, Goncalves, De Los Santos Gomez, et al., 2023). This study has focussed specifically on biochemical epidermal‐dermal crosstalk driven by paracrine signalling, as one of multiple mechanisms that contribute to the complex ageing phenotype. However, it is well established that direct cellular contact and physical interactions through mechanotransduction and alterations in tissue topograpahy, that is, rete ridge/dermal papilla morphology significantly impacts cellular behaviour, which was outside the scope of this study. Innovative strides made in the field of skin equivalent technologies that incorporate rete ridge structure in vitro, would enable future studies to probe this relationship in vitro (Admane et al., 2019; Nagarajan et al., 2023; Shen et al., 2021). The use of HSEs described in this study as a tool to isolate the intricacies of the multifactorial process that is skin ageing, is an pioneering step to uncovered the fundamental molecular processes involved in epidermal‐dermal communication. However, this study is a foundation upon which complexity can be increased to expand our knowledge of the underlying biological pathways involved in skin ageing, in‐line with technological advancements.

The use of the EO‐HSE system allowed us to study the impact of diffusible factors secreted by fibroblasts on an already established epidermal tissue, akin to in vivo, as the epidermal construct was allowed 7 days to form before addition of conditioned medium. This is contrasted by the FT‐HSEs that demonstrates the impact of ageing fibroblasts on epidermal development, and offers a more complex viewpoint through a more physiologically relevant microenvironment encompassing basement membrane formation, endogenous ECM composition and structural/direct interactions between the two compartments. Overall, both in vitro systems offer insight into the cellular and molecular processes that underpin dermal‐epidermal crosstalk during ageing and the use of both in tandem, allows us to elucidate and isolate specific mechanistic activity.

Fibroblasts are known to secrete diffusible factors, capable of impacting the epidermis, such as KGF and GM‐CSF (Maas‐Szabowski et al., 1999; Szabowski et al., 2000) however, the paracrine impact of dermal ageing on epidermal structure, has not been widely discussed. Inflammageing and the development of SASP are well documented phenomena that occur as skin ages (Jarrold et al., 2022; Pilkington et al., 2021; Yue et al., 2022). Here, we describe the induction of a pro‐inflammatory phenotype in ageing fibroblast populations, resulting in the secretion of pro‐inflammatory cytokines, and propose this as a potential mechanism of action.

Secretion of inflammatory cytokines such as TNF‐α, a key mediator in the epidermal response to pathological conditions, chemical irritation, bacterial infection and UV radiation, was significantly increased in the ageing fibroblast population (Juráňová et al., 2017). Other pro‐inflammatory mediators found to be increasingly secreted by ageing fibroblasts such as IL‐8 and MCP‐1 are often upregulated following UVB exposure and may perhaps be a consequence of photoageing (Kang et al., 2007). Interestingly, anti‐inflammatory IL‐10 was also significantly increased in the ageing population, expression of which has been closely linked to collagenase expression and is associated with ECM breakdown and remodelling, a hallmark of the ageing process (Reitamo et al., 1994). Furthermore IL‐13, associated with atopic dermatitis pathogenesis was also upregulated in an ageing fibroblast population, which has been linked to barrier function impairment (Bieber, 2020).

We postulate the link between a changing dermal secretome, promoting the secretion of pro‐inflammatory mediators during the ageing process, is a crucial driver responsible for changes in keratinocyte morphology associated with an ageing phenotype, such as the reduction in height and increase in width observed in this study. Further studies could apply exogenous cytokines onto EO‐HSEs to confirm if the observations described herein are recapitulated, and also use of receptor antagonism to recover phenotypic changes reported in this study. This would corroborate the findings described in this study and provide further evidence as to the contribution of an ageing dermis on the overlying epidermal tissue. This would allow us to achieve both a reductionist and complex approach, to investigate the cellular response to single cytokines and in combination to determine if phenotypic changes are driven by a balance of multiple cytokines (Wang & Plikus, 2024).

In this study, we describe the application of bioengineered skin constructs to probe the molecular events that govern communication between the dermis and epidermis during skin ageing. We demonstrate specific changes to epidermal morphology, such as epidermal thinning, appear to be driven by changes in keratinocyte shape rather than a significant reduction in cell proliferation. Although this study is limited in the use of 1 donor cell line per age group, it does support previous findings reported in the literature such as changes in epidermal structure and epidermal thinning, observed in other bioengineered skin equivalent systems (Costello et al., 2022). These changes are induced by paracrine mechanisms as demonstrated through the monoculture of EO‐HSEs with ageing fibroblast conditioned medium and is associated with an increase in release of pro‐inflammatory cytokines from ageing fibroblasts, and are particularly stark as the keratinocytes used to create the EO‐HSEs are neonatal in origin. This demonstrates the ability of conditioned medium from ageing fibroblasts to induce morphological changes in neonatal keratinocytes, the same cell population that was used for both conditions, controlling for biological variability and age‐associated changes independent of fibroblast condition.

Not only does this study provide insight into the molecular changes that occur in the dermis during ageing, but also provides a platform for further investigations. The use of HSE technologies to investigate and recapitulate aspects of ageing, can be applied to a range of pursuits including: active screening, fundamental insights, identification of novel signalling pathways, transcriptomic analysis and wound healing studies. Furthermore, we have previously demonstrated the incorporation of melanocytes within the FT‐HSE (Goncalves et al., 2023), which would allow for the investigation into the impact of dermal ageing on skin pigmentation.

## AUTHOR CONTRIBUTION

Lydia Costello designed the study, conducted the experiments. Lydia Costello & Kirsty Goncalves wrote the original manuscript which was reviewed by all authors. Kirsty Goncalves & Paola De Los Santos Gomez helped with samples analysis. Ben Hulette, Teresa Dicolandrea, John Oblong, Robert Isfort, Michael J. Flagler and Charlie Bascom provided valuable feedback on data analysis. Stefan Przyborski is the Principal Investigator of this project. All co‐authors read and approved the final manuscript.

## CONFLICT OF INTEREST STATEMENT

Ben Hulette, Teresa Dicolandrea, Robert Isfort, John Oblong, Michael J.Flagler, Charlie Bascom were full‐time employees of Procter & Gamble (Cincinnati, OH, USA) at the time this study was conducted. This work was supported by funding from The Procter & Gamble Company. Stefan Przyborski collaborates and acts as a technical consultant for the company Reprocell Europe Ltd. All other authors declare no conflicts of interest.

## ETHICS STATEMENT

Ethical approval was not required for this study because only commercially available established cell lines were used.

## Supporting information

Supporting information.

## References

[jcp31463-bib-0001] Admane, P. , Gupta, A. C. , Jois, P. , Roy, S. , Chandrasekharan Lakshmanan, C. , Kalsi, G. , Bandyopadhyay, B. , & Ghosh, S. (2019). Direct 3D bioprinted full‐thickness skin constructs recapitulate regulatory signaling pathways and physiology of human skin. Bioprinting, 15, e00051. 10.1016/j.bprint.2019.e00051

[jcp31463-bib-0002] Allcock, B. , Wei, W. , Goncalves, K. , Hoyle, H. , Robert, A. , Quelch‐Cliffe, R. , Hayward, A. , Cooper, J. , & Przyborski, S. (2023). Impact of the physical cellular microenvironment on the structure and function of a model hepatocyte cell line for drug toxicity applications. Cells, 12(Issue 19), 2408. 10.3390/cells12192408 37830622 PMC10572302

[jcp31463-bib-0003] Bieber, T. (2020). Interleukin‐13: targeting an underestimated cytokine in atopic dermatitis. Allergy, 75(1), 54–62. 10.1111/all.13954 31230370

[jcp31463-bib-0004] Billingham, R. E. , & Silvers, W. K. (1967). Studies on the conservation of epidermal specificities of skin and certain mucosas in adult mammals. The Journal of Experimental Medicine, 125(3), 429–446. 10.1084/jem.125.3.429 5334545 PMC2138290

[jcp31463-bib-0005] Bjerke, D. L. , Li, R. , Price, J. M. , Dobson, R. L. M. , Rodrigues, M. , Tey, C. , Vires, L. , Adams, R. L. , Sherrill, J. D. , Styczynski, P. B. , Goncalves, K. , Maltman, V. , Przyborski, S. , & Oblong, J. E. (2021). The vitamin A ester retinyl propionate has a unique metabolic profile and higher retinoid‐related bioactivity over retinol and retinyl palmitate in human skin models. Experimental Dermatology, 30(2), 226–236. 10.1111/exd.14219 33098193

[jcp31463-bib-0006] Coppé, J.‐P. , Desprez, P.‐Y. , Krtolica, A. , & Campisi, J. (2010). The senescence‐associated secretory phenotype: the dark side of tumor suppression. Annual Review of Pathology: Mechanisms of Disease, 5, 99–118. 10.1146/annurev-pathol-121808-102144 PMC4166495 20078217

[jcp31463-bib-0007] Coppé, J.‐P. , Patil, C. K. , Rodier, F. , Sun, Y. , Muñoz, D. P. , Goldstein, J. , Nelson, P. S. , Desprez, P.‐Y. , & Campisi, J. (2008). Senescence‐associated secretory phenotypes reveal cell‐nonautonomous functions of oncogenic RAS and the p53 tumor suppressor. PLoS Biology, 6(12), e301. 10.1371/journal.pbio.0060301 19053174 PMC2592359

[jcp31463-bib-0008] Costello, L. , Dicolandrea, T. , Tasseff, R. , Isfort, R. , Bascom, C. , von Zglinicki, T. , & Przyborski, S. (2022). Tissue engineering strategies to bioengineer the ageing skin phenotype in vitro. Aging cell, 21(2), e13550. 10.1111/acel.13550 35037366 PMC8844123

[jcp31463-bib-0009] Costello, L. , Fullard, N. , Roger, M. , Bradbury, S. , Dicolandrea, T. , Isfort, R. , Bascom, C. , & Przyborski, S. (2019). Engineering a multilayered skin equivalent: the importance of endogenous extracellular matrix maturation to provide robustness and reproducibility. Methods in Molecular Biology (Clifton, N.J.), 1993, 107–122. 10.1007/978-1-4939-9473-1_9 31148082

[jcp31463-bib-0010] Costello, L. , Goncalves, K. , De Los Santos Gomez, P. , Simpson, A. , Maltman, V. , Ritchie, P. , Tasseff, R. , Isfort, R. , Dicolandrea, T. , Wei, X. , Määttä, A. , Karakesisoglou, I. , Markiewicz, E. , Bascom, C. C. , & Przyborski, S. (2023). Quantitative morphometric analysis of intrinsic and extrinsic skin ageing in individuals with fitzpatrick skin types II–III. Experimental Dermatology, 32(n/a), 620–631. 10.1111/exd.14754 36695185 PMC10947487

[jcp31463-bib-0011] Costello, L. , Goncalves, K. , Maltman, V. , Barrett, N. , Shah, K. , Stephens, A. , Dicolandrea, T. , Ambrogio, I. , Hodgson, E. , & Przyborski, S. (2023). Development of a novel in vitro strategy to understand the impact of shaving on skin health: combining tape strip exfoliation and human skin equivalent technology. In Frontiers in Medicine, 10, 1236790. https://www.frontiersin.org/articles/10.3389/fmed.2023.1236790 10.3389/fmed.2023.1236790 PMC10652890 38020123

[jcp31463-bib-0012] Coulomb, B. , Lebreton, C. , & Dubertret, L. (1989). Influence of human dermal fibroblasts on epidermalization. Journal of Investigative Dermatology, 92(1), 122–125. 10.1111/1523-1747.ep13071335 2909624

[jcp31463-bib-0013] Farage, M. A. , Miller, K. W. , Elsner, P. , & Maibach, H. I. (2013). Characteristics of the aging skin. Advances in Wound Care, 2(1), 5–10. 10.1089/wound.2011.0356 24527317 PMC3840548

[jcp31463-bib-0014] Freer, M. , Darling, N. , Goncalves, K. , Mills, K. J. , & Przyborski, S. (2023). Development of a mammalian neurosensory full‐thickness skin equivalent and its application to screen sensitizing stimuli. Bioengineering & Translational Medicine, 8(n/a), e10484. 10.1002/btm2.10484 37206205 PMC10189474

[jcp31463-bib-0015] Goncalves, K. , De Los Santos Gomez, P. , Costello, L. , Smith, L. , Mead, H. , Simpson, A. , & Przyborski, S. (2023). Investigation into the effect of skin tone modulators and exogenous stress on skin pigmentation utilizing a novel bioengineered skin equivalent. Bioengineering & Translational Medicine, 8(2), e10415. 10.1002/btm2.10415 36925688 PMC10013773

[jcp31463-bib-0016] Hausmann, C. , Zoschke, C. , Wolff, C. , Darvin, M. E. , Sochorová, M. , Kováčik, A. , Wanjiku, B. , Schumacher, F. , Tigges, J. , Kleuser, B. , Lademann, J. , Fritsche, E. , Vávrová, K. , Ma, N. , & Schäfer‐Korting, M. (2019). Fibroblast origin shapes tissue homeostasis, epidermal differentiation, and drug uptake. Scientific Reports, 9(1), 2913. 10.1038/s41598-019-39770-6 30814627 PMC6393472

[jcp31463-bib-0017] Jarrold, B. B. , Tan, C. Y. R. , Ho, C. Y. , Soon, A. L. , Lam, T. T. , Yang, X. , Nguyen, C. , Guo, W. , Chew, Y. C. , DeAngelis, Y. M. , Costello, L. , De Los Santos Gomez, P. , Przyborski, S. , Bellanger, S. , Dreesen, O. , Kimball, A. B. , & Oblong, J. E. (2022). Early onset of senescence and imbalanced epidermal homeostasis across the decades in photoexposed human skin: fingerprints of inflammaging. Experimental Dermatology, 31(11), 1748–1760. 10.1111/exd.14654 36320153

[jcp31463-bib-0018] Juráňová, J. , Franková, J. , & Ulrichová, J. (2017). The role of keratinocytes in inflammation. Journal of Applied Biomedicine, 15(3), 169–179. 10.1016/j.jab.2017.05.003

[jcp31463-bib-0019] Kang, J. S. , Kim, H. N. , Jung, D. J. , Kim, J. E. , Mun, G. H. , Kim, Y. S. , Cho, D. , Shin, D. H. , Hwang, Y.‐I. , & Lee, W. J. (2007). Regulation of UVB‐Induced IL‐8 and MCP‐1 production in skin keratinocytes by increasing vitamin C uptake via the redistribution of SVCT‐1 from the cytosol to the membrane. Journal of Investigative Dermatology, 127(3), 698–706. 10.1038/sj.jid.5700572 17008880

[jcp31463-bib-0020] Kratochwil, K. , & Yamada, K. M. (1983). Embryonic induction. In K. M. Yamada *Editor*, Cell Interactions and Development. Molecular Mechanisms (pp. 100–122). John Wiley and Sons.

[jcp31463-bib-0021] Lacroix, S. , Bouez, C. , Vidal, S. , Cenizo, V. , Reymermier, C. , Justin, V. , Vičanová, J. , & Damour, O. (2007). Supplementation with a complex of active nutrients improved dermal and epidermal characteristics in skin equivalents generated from fibroblasts from young or aged donors. Biogerontology, 8(2), 97–109. 10.1007/s10522-006-9037-7 17028931

[jcp31463-bib-0022] Langton, A. K. , Alessi, S. , Hann, M. , Chien, A. L. , Kang, S. , Griffiths, C. , & Watson, R. (2019). Aging in skin of color: disruption to elastic fiber organization is detrimental to skin's biomechanical function. The Journal of Investigative Dermatology, 139(4), 779–788. 10.1016/j.jid.2018.10.026 30404021

[jcp31463-bib-0023] Langton, A. K. , Graham, H. K. , Griffiths, C. E. M. , & Watson, R. E. B. (2019). Ageing significantly impacts the biomechanical function and structural composition of skin. Experimental Dermatology, 28(8), 981–984. 10.1111/exd.13980 31152614 PMC6851988

[jcp31463-bib-0024] Lee, D. H. , Oh, J.‐H. , & Chung, J. H. (2016). Glycosaminoglycan and proteoglycan in skin aging. Journal of Dermatological Science, 83(3), 174–181. 10.1016/j.jdermsci.2016.05.016 27378089

[jcp31463-bib-0025] Maas‐Szabowski, N. , Shimotoyodome, A. , & Fusenig, N. E. (1999). Keratinocyte growth regulation in fibroblast cocultures via a double paracrine mechanism. Journal of Cell Science, 112(12), 1843–1853. 10.1242/jcs.112.12.1843 10341204

[jcp31463-bib-0026] Mackenzie, I. C. , & Fusenig, N. E. (1983). Regeneration of organized epithelial structure. Journal of Investigative Dermatology, 81(1 Suppl.), S189–S194. 10.1111/1523-1747.ep12541093 6863990

[jcp31463-bib-0027] Marcos‐Garcés, V. , Molina Aguilar, P. , Bea Serrano, C. , García Bustos, V. , Benavent Seguí, J. , Ferrández Izquierdo, A. , & Ruiz‐Saurí, A. (2014). Age‐related dermal collagen changes during development, maturation and ageing ‐ a morphometric and comparative study. Journal of Anatomy, 225(1), 98–108. 10.1111/joa.12186 24754576 PMC4089350

[jcp31463-bib-0028] Nagarajan, M. B. , Ainscough, A. J. , Reynolds, D. S. , Uzel, S. G. M. , Bjork, J. W. , Baker, B. A. , McNulty, A. K. , Woulfe, S. L. , & Lewis, J. A. (2023). Biomimetic human skin model patterned with rete ridges. Biofabrication, 16(1), 015006. 10.1088/1758-5090/acfc29 37734324

[jcp31463-bib-0029] Nelson, G. , Kucheryavenko, O. , Wordsworth, J. , & von Zglinicki, T. (2018). The senescent bystander effect is caused by ROS‐activated NF‐κB signalling. Mechanisms of Ageing and Development, 170, 30–36. 10.1016/j.mad.2017.08.005 28837845 PMC5861994

[jcp31463-bib-0030] Nelson, G. , Wordsworth, J. , Wang, C. , Jurk, D. , Lawless, C. , Martin‐Ruiz, C. , & von Zglinicki, T. (2012). A senescent cell bystander effect: senescence‐induced senescence. Aging cell, 11(2), 345–349. 10.1111/j.1474-9726.2012.00795.x 22321662 PMC3488292

[jcp31463-bib-0031] Passos, J. F. , Nelson, G. , Wang, C. , Richter, T. , Simillion, C. , Proctor, C. J. , Miwa, S. , Olijslagers, S. , Hallinan, J. , Wipat, A. , Saretzki, G. , Rudolph, K. L. , Kirkwood, T. B. L. , & von Zglinicki, T. (2010). Feedback between p21 and reactive oxygen production is necessary for cell senescence. Molecular Systems Biology, 6, 347. 10.1038/msb.2010.5 20160708 PMC2835567

[jcp31463-bib-0032] Pennacchi, P. C. , de Almeida, M. E. S. , Gomes, O. L. A. , Faião‐Flores, F. , de Araújo Crepaldi, M. C. , Dos Santos, M. F. , de Moraes Barros, S. B. , & Maria‐Engler, S. S. (2015). Glycated reconstructed human skin as a platform to study the pathogenesis of skin aging. Tissue engineering. Part A, 21(17–18), 2417–2425. 10.1089/ten.TEA.2015.0009 26132636

[jcp31463-bib-0033] Pilkington, S. M. , Bulfone‐Paus, S. , Griffiths, C. E. M. , & Watson, R. E. B. (2021). Inflammaging and the skin. Journal of Investigative Dermatology, 141(4S), 1087–1095. 10.1016/j.jid.2020.11.006 33358020

[jcp31463-bib-0034] Quan, T. (2023). Molecular insights of human skin epidermal and dermal aging. Journal of Dermatological Science, 112, 48–53. 10.1016/j.jdermsci.2023.08.006 37661473 PMC13155249

[jcp31463-bib-0035] Reitamo, S. , Remitz, A. , Tamai, K. , & Uitto, J. (1994). Interleukin‐10 modulates type I collagen and matrix metalloprotease gene expression in cultured human skin fibroblasts. Journal of Clinical Investigation, 94(6), 2489–2492. 10.1172/JCI117618 7989607 PMC330082

[jcp31463-bib-0036] Roger, M. , Fullard, N. , Costello, L. , Bradbury, S. , Markiewicz, E. , O'Reilly, S. , Darling, N. , Ritchie, P. , Määttä, A. , Karakesisoglou, I. , Nelson, G. , von Zglinicki, T. , Dicolandrea, T. , Isfort, R. , Bascom, C. , & Przyborski, S. (2019). Bioengineering the microanatomy of human skin. Journal of Anatomy, 234(4), 438–455. 10.1111/joa.12942 30740672 PMC6422806

[jcp31463-bib-0037] Schneider, C. A. , Rasband, W. S. , & Eliceiri, K. W. (2012). NIH image to ImageJ: 25 years of image analysis. Nature Methods, 9(7), 671–675. 10.1038/nmeth.2089 22930834 PMC5554542

[jcp31463-bib-0038] Shen, Z. , Cao, Y. , Li, M. , Yan, Y. , Cheng, R. , Zhao, Y. , Shao, Q. , Wang, J. , & Sang, S. (2021). Construction of tissue‐engineered skin with rete ridges using co‐network hydrogels of gelatin methacrylated and poly(ethylene glycol) diacrylate. Materials Science & Engineering, C: Materials for Biological Applications, 129, 112360. 10.1016/j.msec.2021.112360 34579879

[jcp31463-bib-0039] Szabowski, A. , Maas‐Szabowski, N. , Andrecht, S. , Kolbus, A. , Schorpp‐Kistner, M. , Fusenig, N. E. , & Angel, P. (2000). c‐Jun and JunB antagonistically control cytokine‐regulated mesenchymal‐epidermal interaction in skin. Cell, 103(5), 745–755. 10.1016/s0092-8674(00)00178-1 11114331

[jcp31463-bib-0400] United Nations. (2015). World population ageing 2015 . https://www.un.org/en/development/desa/population/publications/pdf/ageing/WPA2015_Report.pdf

[jcp31463-bib-0040] Varani, J. , Dame, M. K. , Rittie, L. , Fligiel, S. E. G. , Kang, S. , Fisher, G. J. , & Voorhees, J. J. (2006). Decreased collagen production in chronologically aged skin. The American Journal of Pathology, 168(6), 1861–1868. 10.2353/ajpath.2006.051302 16723701 PMC1606623

[jcp31463-bib-0041] Waldera Lupa, D. M. , Kalfalah, F. , Safferling, K. , Boukamp, P. , Poschmann, G. , Volpi, E. , Götz‐Rösch, C. , Bernerd, F. , Haag, L. , Huebenthal, U. , Fritsche, E. , Boege, F. , Grabe, N. , Tigges, J. , Stühler, K. , & Krutmann, J. (2015). Characterization of skin Aging‐Associated secreted proteins (SAASP) produced by dermal fibroblasts isolated from intrinsically aged human skin. Journal of Investigative Dermatology, 135(8), 1954–1968. 10.1038/jid.2015.120 25815425

[jcp31463-bib-0042] Wang, X. , & Plikus, M. V. (2024). Aged skin cells nurture stem cells toward regeneration. Journal of Investigative Dermatology, 144(1), 11–14. 10.1016/j.jid.2023.07.028 37897482

[jcp31463-bib-0043] Wessells, N. K. (1962). Tissue interactions during skin histodifferentiation. Developmental Biology, 4, 87–107. 10.1016/0012-1606(62)90034-9 14006278

[jcp31463-bib-0044] Wheeler, C. E. , & Briggaman, R. A. (1971). Epidermal‐dermal interactions in adult human skin. II. the nature of the dermal influence. Journal of Investigative Dermatology, 56(1), 18–26. 10.1111/1523-1747.ep12291866 4933754

[jcp31463-bib-0045] Yue, Z. , Nie, L. , Zhao, P. , Ji, N. , Liao, G. , & Wang, Q. (2022). Senescence‐associated secretory phenotype and its impact on oral immune homeostasis. In Frontiers in Immunology, 13, 1019313. https://www.frontiersin.org/articles/10.3389/fimmu.2022.1019313 10.3389/fimmu.2022.1019313 PMC9581398 36275775

