# Supplementary material for: Investigation into the significant role of dermal‐epidermal interactions in skin ageing utilising a bioengineered skin construct
Source: J Cell Physiol. 2024 Oct 8;240(1):e31463. doi: 10.1002/jcp.31463 (PMC11701872; doi:10.1002/jcp.31463)
Supplement: Supplementary file 1 — Supporting information. [file JCP-240-0-s001.docx]

| **Antibody** | **Supplier** | **Product Code** | **Dilution** |
| --- | --- | --- | --- |
| Collagen I | Abcam | ab34710 | 1:100 |
| Collagen III | Abcam | ab7778 | 1:100 |
| p63 | Abcam | ab12462 | 1:100 |
| ki67 | Abcam | ab16667 | 1:500 |
| Cytokeratin-14 (K14) | Abcam | ab7800 | 1:100 |
| Cytokeratin-10 (K10) | Abcam | ab76318 | 1:100 |
| Filaggrin | Abcam | ab17808 | 1:100 |
| Integrin α6 | Abcam | ab181551 | 1:100 |
| Claudin-1 | Abcam | ab15098 | 1:100 |
| Periplakin | Abcam | ab131269 | 1:100 |
| E-Cadherin | Abcam | ab1416 | 1:100 |
| Involucrin | Abcam | ab53112 | 1:100 |
| Loricrin | Abcam | ab85679 | 1:100 |
| HMGB1 | Abcam | ab79823 | 1:100 |
| Lamin B1 | Abcam | ab108922 | 1:100 |

**Supplementary Table 1 (S1): Primary antibodies used in immunofluorescence staining**
